# Multicenter, prospective, observational study of chemotherapy-induced dysgeusia in gastrointestinal cancer

**DOI:** 10.1007/s00520-022-06936-4

**Published:** 2022-03-15

**Authors:** Ken Ito, Satoshi Yuki, Hiroshi Nakatsumi, Yasuyuki Kawamoto, Kazuaki Harada, Shintaro Nakano, Rika Saito, Takayuki Ando, Kentaro Sawada, Masataka Yagisawa, Atsushi Ishiguro, Masayoshi Dazai, Ichiro Iwanaga, Kazuteru Hatanaka, Atsushi Sato, Ryusuke Matsumoto, Yoshiaki Shindo, Miki Tateyama, Tetsuhito Muranaka, Masaki Katagiri, Isao Yokota, Yuh Sakata, Naoya Sakamoto, Yoshito Komatsu

**Affiliations:** 1 Division of Cancer Center, Hokkaido University Hospital, Sapporo, Japan; 2 Department of Gastroenterology and Hepatology, Hokkaido University Hospital, Sapporo, Japan; 3 Department of Gastroenterology, National Hospital Organization Hokkaido Medical Center, Sapporo, Japan; 4 Third Department of Internal Medicine, University of Toyama, Toyama, Japan; 5 Department of Medical Oncology, Kushiro Rosai Hospital, Kushiro, Japan; 6 Department of Medical Oncology, Japanese Red Cross Kitami Hospital, Kitami, Japan; 7 Department of Medical Oncology, Teine Keijinkai Hospital, Sapporo, Japan; 8 Department of Gastroenterology, Sapporo Medical Center NTT EC, Sapporo, Japan; 9 Department of Medical Oncology, Sapporo Kosei Hospital, Sapporo, Japan; 10 Department of Gastroenterology, Hakodate Municipal Hospital, Hakodate, Japan; 11 Department of Medical Oncology, Hirosaki University Graduate School of Medicine, Hirosaki, Japan; 12 Department of Gastroenterology, Obihiro Kosei Hospital, Obihiro, Japan; 13 Department of Gastroenterological Surgery, Nakadori General Hospital, Akita, Japan; 14 Department of Internal Medicine, Tomakomai Nissho Hospital, Tomakomai, Japan; 15 Department of Gastroenterology, Wakkanai City Hospital, Wakkanai, Japan; 16 Department of Gastroenterology, Sapporo Hokuyu Hospital, Sapporo, Japan; 17 Department of Biostatistics, Graduate School of Medicine, Hokkaido University, Sapporo, Japan; 18 Misawa City Hospital, Misawa, Japan

**Keywords:** Dysgeusia, Taste disorder, Chemotherapy, Gastrointestinal cancer, Polaprezinc, Zinc acetate hydrate

## Abstract

**Purpose:**

Dysgeusia is an adverse event caused by chemotherapy. Although retrospective studies have shown zinc administration improves dysgeusia, there have been no prospective studies. The present study examined effects of zinc therapy on dysgeusia in patients with gastrointestinal cancer.

**Methods:**

This multicenter, prospective, observational study enrolled patients with dysgeusia during chemotherapy treatment. Patients received no intervention (control), polaprezinc p.o., or zinc acetate hydrate p.o., and serum zinc levels were measured at 0 (baseline), 6, and 12 weeks. Dysgeusia was assessed using CTCAE v5.0 and subjective total taste acuity (STTA) criteria using questionnaires at baseline and 12 weeks.

**Results:**

From February 2020 to June 2021, 180 patients were enrolled from 17 institutes. There were no differences in mean baseline serum zinc levels among the groups (67.3, 66.6, and 67.5 μg/dL in the no intervention, polaprezinc, and zinc acetate hydrate groups, respectively. *P* = 0.846). The changes in mean serum zinc levels after 12 weeks were − 3.8, + 14.3, and + 46.6 μg/dL, and the efficacy rates of dysgeusia were 33.3%, 36.8%, and 34.6% using CTCAE and 33.3%, 52.6%, 32.7% using STTA in the no intervention, polaprezinc, and zinc acetate hydrate groups, respectively. The STTA scores improved in all groups, with significant improvement observed in the polaprezinc group compared with the no intervention group (*P* = 0.045).

**Conclusion:**

There was no significant correlation between the degree of serum zinc elevation and improvement in dysgeusia, suggesting that polaprezinc, but not zinc acetate hydrate, was effective in improving chemotherapy-induced dysgeusia.

Trial registration.

UMIN000039653. Date of registration: March 2, 2020.

**Supplementary Information:**

The online version contains supplementary material available at 10.1007/s00520-022-06936-4.

## Introduction

The development of antitumor agents and overall survival in gastrointestinal cancer patients has dramatically improved in the last decade. Increased treatment duration, quality of life (QOL), and management of adverse events has become increasingly important. Diet is an important factor in QOL maintenance, but adverse events of chemotherapy including anorexia, nausea, vomiting, and dysgeusia reduce oral intake [1–4]. Although prevention and treatment have been established for chemotherapy-induced nausea, vomiting, and oral mucositis, there is currently no successful intervention for dysgeusia [5, 6]. The incidence of chemotherapy-induced dysgeusia is around 30–85% [7–17], with moderate-to-severe cases occurring in around 38% of patients [10, 12]. Fluoropyrimidines and platinum-based drugs, which are often used for gastrointestinal cancers, cause dysgeusia in 48.1% and 42.1% of patients, respectively [16]. Long-term dysgeusia may lead to poor nutritional and performance status [1]. Although the precise mechanisms underlying chemotherapy-induced dysgeusia remain unclear, inhibition of differentiation and proliferation of cells in the taste buds via direct toxic action of chemotherapy drugs or neurotoxic effects of chemotherapy may be implicated in the etiology of dysgeusia [7, 18, 19].

Zinc deficiency due to chelation by chemotherapeutic agents may also contribute to the etiology of chemotherapy-induced dysgeusia [4, 18]. High amounts of zinc are required to regenerate the cells in taste buds, indicating that zinc deficiency may cause dysgeusia [20]. Ikeda et al. [21] reported that oral administration of polaprezinc (zinc-l-carnosine) in patients with zinc deficiency or idiopathic dysgeusia increased plasma zinc levels and improved dysgeusia. Therefore, zinc supplementation is likely to be effective in alleviating dysgeusia. However, most studies on the effects of zinc supplementation on chemotherapy-induced dysgeusia have been small retrospective studies [17, 22–25], and there have been no prospective multicenter studies. The relationship between improvement of dysgeusia and zinc supplementation remains unclear. Therefore, we designed a multicenter, prospective observational study to examine the effect of zinc therapy on chemotherapy-induced dysgeusia in patients with gastrointestinal cancer.

## Materials and methods

### Study design and patients

The present investigator-initiated, multicenter, and prospective observational study recruited patients undergoing chemotherapy at 17 institutes in Japan from February 2020 to June 2021. Eligibility criteria included patients presenting with grade ≥ 1 dysgeusia according to the Common Terminology Criteria for Adverse Events (CTCAE) v5.0 [26] or Scale of Subjective Total Taste Acuity (STTA) [27] during chemotherapy for gastrointestinal cancer (including esophageal cancer, gastric cancer, colorectal cancer, pancreatic cancer, and biliary tract cancer). The exclusion criteria were administration of zinc preparations before registration and history of head and neck radiation therapy. The enrolled patients were treated for dysgeusia using one of the following methods: no intervention, zinc acetate hydrate (50–100 mg of zinc per daily normal dose), or polaprezinc (34.1 mg of zinc per daily normal dose). Treatment for dysgeusia was determined by the attending physician. In Japan, many patients with chemotherapy-induced dysgeusia do not receive any therapeutic intervention; therefore, a no intervention group was included in the present study. After registration, physicians collected clinical information related to dysgeusia prospectively for 12 weeks using blood sampling and two questionnaires at the beginning and end of treatment.

### Assessment of serum zinc levels, dysgeusia, QOL, and safety

Serum zinc levels were measured at weeks 0 (enrollment), 6, and 12. Absolute changes in serum zinc levels were calculated by subtracting the value obtained at enrollment from those obtained at weeks 6 and 12 after enrollment. Serum copper levels and nutritional factors, such as hemoglobin and albumin, were also measured at the same time as serum zinc levels, and absolute changes in these were defined in the same way. Vitamin B_12_ levels were also measured at enrollment and after 12 weeks.

Clinical information related to dysgeusia was collected from patients for 12 weeks after registration. Dysgeusia was graded according to the CTCAE v5.0, STTA, Visual Analog Scale (VAS), and Chemotherapy-induced Taste Alteration Scale (CiTAS) criteria [28]. This information was collected twice using questionnaires at enrollment and after 12 weeks. Efficacy rates (%) for CTCAE and STTA scores were calculated by dividing the number of patients whose scores were cured or improved by the total number of patients who participated in each dosage group. QOL was graded according to the quality-of-life questionnaire for cancer patients treated with anticancer drugs (QOL-ACD). This information was included in the dysgeusia questionnaires and collected at enrollment and after 12 weeks. The incidence of adverse effects due to zinc replacement therapy was analyzed statistically.

### Statistical analysis

Absolute changes in the serum zinc levels in each group were compared using Wilcoxon’s signed rank test. Absolute changes in the serum zinc levels of patients in the zinc acetate hydrate and polaprezinc groups relative to the non-intervention group were compared using Steel’s multiple comparison. Improvement in dysgeusia was determined by comparing CTCAE v5.0 and STTA using chi-square test, and the extent of change in VAS and CiTAS was compared using Steel’s multiple comparison test. Change in QOL-ACD was compared using Steel’s multiple comparison test. The objective variable was improvement of dysgeusia, the explanatory variable was the range of change of each factor, and multivariate analysis (multiple regression analysis and logistic regression analysis) was used to investigate the independent factors related to the improvement of dysgeusia, respectively. Among the few small retrospective studies on the efficacy of zinc preparations in chemotherapy-induced dysgeusia, the improvement rate was reported to be around 60–70% [17, 23, 25]. A multicenter, placebo-controlled, double-blind study evaluating the efficacy of zinc preparations in dysgeusia excluding malignancy reported an efficacy rate of 61.9% for zinc preparations compared with 39.5% for placebo [21]. The sample size was set to 60 per group due to feasibility. This size was calculated to be adequate to achieve a statistical power of 80% with significance level of 5% under the improvement rates of 65% and 40% in dysgeusia with zinc preparation and no treatment, respectively. Statistical analyses were performed using JMP 14 (SAS Institute Inc., Cary, NC, USA). All *P*-values > 0.05 were considered significant.

## Results

### Patient characteristics

The enrolled institutions and the number of patients enrolled at each institution are shown in Online Resource 1. Among a total of 180 patients enrolled in the study, five were excluded due to noncompliance with the participation criteria (four patients had grade 0 CTCAE and STTA, and one had neuroendocrine carcinoma), and three patients in the no intervention group began receiving zinc therapy. Thus, the final total of patients eligible for evaluation in the present study included 53 in the no intervention group, 60 in the zinc acetate hydrate group, and 59 in the polaprezinc group. The characteristics of the 172 patients who participated in the study are summarized in Table 1. There were more females in the no intervention group (male/female: 25/28) and more males in the zinc acetate hydrate and polaprezinc groups (male/female: 42/18, 26/23, respectively). The median age of the polaprezinc group was 67 years, which was slightly younger than that of the no intervention and zinc acetate hydrate groups, which was 70 years. The mean serum zinc levels at baseline were 67.3 μg/dL (95% confidence interval (CI), 63.0–71.6 μg/dL) in the no intervention group, 67.5 μg/dL (95% CI, 63.9–71.1 μg/dL) in the zinc acetate hydrate group, and 66.6 μg/dL (95% CI, 63.0–70.2 μg/dL) in the polaprezinc group. The mean serum zinc levels at baseline were similar in each group and below the normal limit of 80 μg/dL.

**Table 1:** Patient characteristics before zinc supplementation

**n (%)**		**No intervention (*n* = 53)**	**Zinc acetate hydrate (*n* = 60)**	**Polaprezinc (*n* = 59)**
Sex	Male Female	25 (47) 28 (53)	42 (70) 18 (30)	36 (61) 23 (39)
Age	Median (range)	70 (27–86)	70 (42–81)	67 (40–79)
Body mass index	Median (range)	21.0 (14.6–33.5)	21.9 (16.2–30.7)	21.9 (15.4–30.5)
ECOG PS	0 1 2	28 (53) 24 (45) 1 (2)	37 (62) 23 (38) 0 (0)	35 (59) 24 (41) 0 (0)
Professional oral care^*^	Yes No	18 (34) 35 (66)	26 (43) 34 (57)	20 (34) 39 (66)
Mouth rinses	Yes No	27 (51) 26 (49)	39 (65) 21 (35)	32 (54) 27 (46)
Duration of dysgeusia (day)	Median (range)	36 (1–1.933)	23 (1–1.167)	29 (1–3.697)
Cancer site	Esophagus Gastric Colorectal Pancreatic Biliary tract Others	2 (4) 15 (28) 26 (49) 6 (11) 4 (8) 0 (0)	1 (2) 12 (20) 28 (47) 12 (20) 6 (10) 1 (2)	0 (0) 14 (24) 30 (51) 7 (12) 7 (12) 1 (2)
Chemotherapy drugs	Platinum Fluoropyrimidines Taxanes	28 (53) 38 (72) 10 (19)	30 (50) 48 (80) 8 (13)	35 (59) 45 (76) 7 (12)
Number of pretreatments	0 1 ≥ 2	37 (70) 11 (21) 5 (9)	41 (68) 17 (28) 2 (3)	41 (69) 12 (20) 6 (10)
Serum zinc (μg/dL)	Median (range)	68/(29–113)	64 / (43–109)	66 (25–109)
Hb (g/dL)	Median (range)	11.2 (8.3–16.1)	11.5 (8.1–16.4)	11.0 (8.0–16.2)
Alb (g/dL)	Median (range)	3.6 (2.1–4.5)	3.7 (2.6–4.5)	3.5 (2.4–4.5)
Ferritin (ng/mL)	Median (range)	129.5 (3.7–1855)	155.1 (7–1343)	97.2 (4–1051)
Vit B_12_ (pg/mL)	Median (range)	457 (110–1500)	478 (108–1500)	513 (144–1500)

*Alb* albumin, *Hb* hemoglobin, *ECOG PS* Eastern Cooperative Oncology Group performance score

^*^Oral care delivered by dental professionals

### Zinc supplementation

Zinc supplementation was successfully achieved for 12 weeks by 53 patients in the polaprezinc group and 46 patients in the zinc acetate hydrate group. The reasons for discontinuation were mostly unrelated to zinc therapy and included exacerbation of the primary disease (four patients in each group), cholangitis (two patients in the zinc acetate hydrate group), self-decision (two patients in the polaprezinc group and six patients in the zinc acetate hydrate group), or physician’s opinion (one patient in zinc acetate hydrate group). Discontinuation due to chest discomfort related to zinc supplementation was observed in one patient in the zinc acetate hydrate group. The mean number of days on medication for patients who discontinued was 57.8 days (95% CI, 39.9–75.7) in the polaprezinc group and 34.1 days (95% CI, 24.8–43.5) in the zinc acetate hydrate group.

### Changes in serum zinc levels

**Fig. 1:**
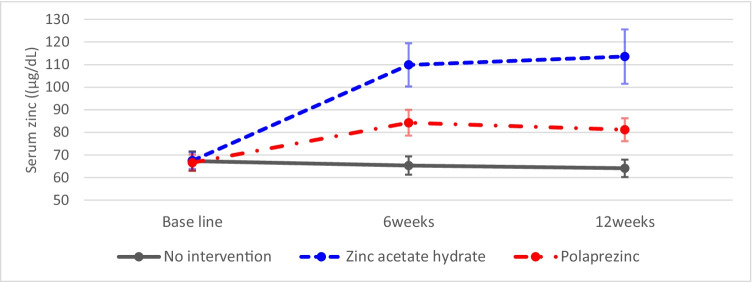
Mean serum zinc levels determined at each evaluation time point

The mean serum zinc levels at each evaluation time point are shown in Fig. 1. In the zinc acetate hydrate and polaprezinc groups, there was a significant increase in serum zinc levels at 6 weeks (*P* < 0.001, respectively; Wilcoxon’s signed rank test), which was maintained until 12 weeks. The mean changes in serum zinc levels at 12 weeks in the no intervention, zinc acetate, and polaprezinc groups were − 3.8 μg/dL (95% CI, − 8.7–1.1 μg/dL), 46.6 μg/dL (95% CI, 34.5–58.7 μg/dL), and 14.3 μg/dL (95% CI, 9.0–19.7 μg/dL), respectively. These results clearly demonstrate that serum zinc levels increased in a dose-dependent manner, and the increase in serum zinc in the group receiving zinc acetate hydrate and polaprezinc was statistically significant compared with that of the no intervention group (*P* < 0.001; Steel’s multiple comparison test).

### Changes in dysgeusia score

**Fig. 2:**
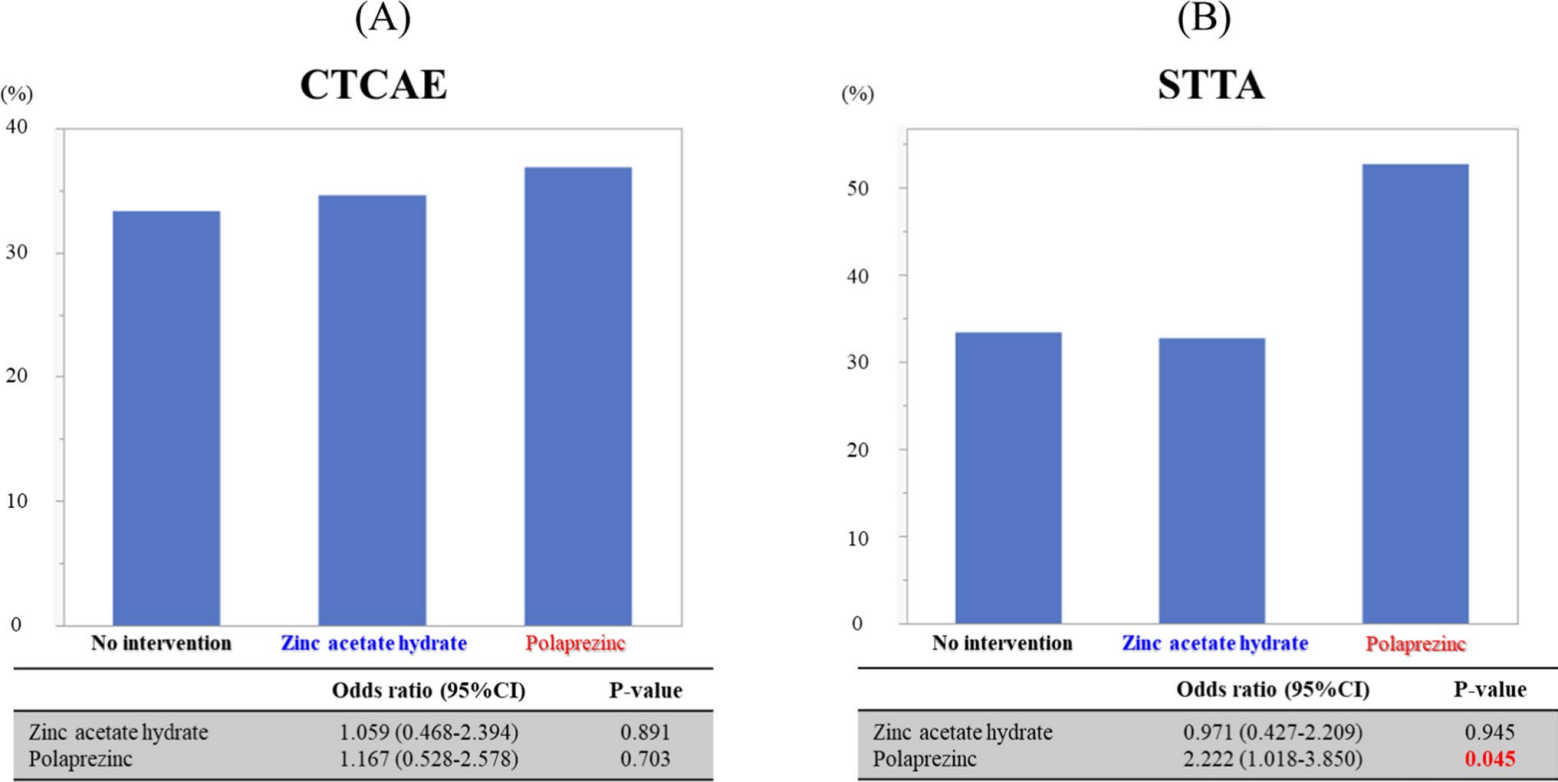
Efficacy rates (%) for CTCAE and STTA. **A** CTCAE and **B** STTA. *P*-value was calculated using logistic regression analysis

Changes in dysgeusia determined using CTCAE and STTA score are shown in Fig. 2. At week 12, a total of 2, 8, and 2 patients were missing questionnaires in the no intervention, zinc acetate hydrate, and polaprezinc groups, respectively. The efficacy rate of the STTA score was 33.3% (17/51), 32.7% (17/52), and 52.6% (30/57) in the no intervention, zinc acetate hydrate, and polaprezinc groups, respectively, and there was no statistically significant difference between the groups (*P* = 0.053; chi-square test). The odds ratios of zinc acetate hydrate and polaprezinc to no intervention were 0.971 (95% CI, 0.427–2.209; *P* = 0.945) and 2.222 (95% CI, 1.018–3.850; *P* = 0.045). There was a statistically significant improvement in STTA score in the polaprezinc group compared with the no intervention group, but no statistical difference in the zinc acetate hydrate group compared with the no intervention group. The efficacy rates using the CTCAE score were 33.3% (17/51), 34.6% (18/52), and 36.8% (21/57) in the no intervention, zinc acetate hydrate, and polaprezinc groups, respectively, and there were no statistically significant differences between the groups (*P* = 0.927; chi-square test). The odds ratios of zinc acetate hydrate and polaprezinc compared with no intervention were 1.059 (95% CI, 0.468–2.394; *P* = 0.891) and 1.167 (95% CI, 0.528–2.578; *P* = 0.703), respectively. Changes in dysgeusia determined using VAS are shown in Online Resource 2. The mean changes in VAS score were 6.6 mm (95% CI, 1.0–12.1), 6.1 mm (95% CI, − 1.6–13.7), and 10.8 mm (95% CI, 4.7–16.9) in the no intervention, zinc acetate hydrate, and polaprezinc groups, respectively, and there were no statistically significant differences between the zinc acetate hydrate and polaprezinc groups compared with the no intervention group (*P* = 0.994, *P* = 0.669, respectively; Steel’s multiple comparison test). Changes in taste perception measured using CiTAS are shown in Online Resource 3. The mean changes of decline in basic taste using the CiTAS subscore were 0.16 (95% CI, − 0.04–0.37), 0.36 (95% CI, 0.05–0.67), and 0.47 (95% CI, 0.26–0.68) in the no intervention, zinc acetate hydrate, and polaprezinc groups, respectively. The mean changes in discomfort determined using the CiTAS subscore were − 0.03 (95% CI, − 0.21–0.15), 0.12 (95% CI, − 0.09–0.33), and 0.22 (95% CI, 0.04–0.40); phantogeusia and parageusia were 0.03 (95% CI, − 0.16–0.22), 0.13 (95% CI, − 0.21–0.47) and 0.21 (95% CI, − 0.06–0.48), and general taste alterations were 0.08 (95% CI, − 0.09–0.26), 0.35 (95% CI, 0.04–0.65), and 0.44 (95% CI, 0.18–0.71) in the no intervention, zinc acetate hydrate, and polaprezinc groups, respectively. There were no statistically significant differences between the groups. However, CTCAE, VAS, and CiTAS scores showed a trend toward improvement in dysgeusia in the polaprezinc group, but not the zinc acetate hydrate group, compared with the no intervention group.

### Factors related to improvement of dysgeusia

Multivariate analysis was conducted using multiple regression analysis and logistic regression analysis to investigate independent factors related to improvement of dysgeusia. The results of logistic regression analysis for STTA are shown in Online Resource 4. Polaprezinc was analyzed as an independent significant factor for improvement in taste disorder (*P* = 0.013).

### Quality of life

The results of the QOL assessment by QOL-ACD are shown in Table 2. There was a significant improvement in psychological condition of QOL-ACD subdomain in the polaprezinc group compared with the no intervention group. There was a significant improvement in face scale in the QOL-ACD subdomain in the zinc acetate hydrate group compared with the no intervention group. However, in the other subdomains, there was no significant improvement in the zinc-treated group compared with the non-intervention group, and there was no significant improvement in total score. Therefore, QOL was not improved by zinc administration.

**Table 2:** The results of QOL assessment using QOL-ACD

Average (95% CI)	**No intervention (*n* = 51)**	**Zinc acetate hydrate (*n* = 52)**		**Polaprezinc (*n* = 57)**	
			***P value***^†^		***P value***^†^
Daily activity	− 1.41 (− 2.80– − 0.02)	− 0.50 (− 1.77–0.77)	0.686^*^	0.00 (− 1.37–1.37)	0.414^*^
Physical condition	0.08 (− 1.11–1.27)	0.46 (− 0.64–1.57)	0.938^*^	1.12 (− 0.02–2.26)	0.454^*^
Psychological condition	− 0.63 (− 1.66–0.41)	0.19 (− 0.45–0.84)	0.360^*^	1.12 (0.10–2.14)	0.049^*^
Social attitude	0.14 (− 0.91–1.19)	0.63 (− 0.27–1.54)	0.817^*^	0.54 (− 0.42–1.51)	0.921^*^
Face scale	− 0.31 (− 0.60– − 0.02)	0.19 (− 0.04–0.42)	0.021^*^	0.04 (− 0.21–0.28)	0.190^*^
Total	− 2.14 (− 5.50–1.22)	0.98 (− 1.64–3.60)	0.237^*^	2.82 (− 0.36–6.01)	0.083^*^

*QOL* quality of life, *QOL-ACD* quality-of-life questionnaire for cancer patients treated with anticancer drugs, *CI* confidence interval

^*^Steel’s multiple comparison test

^†^vs no intervention

## Discussion

This is the first large-scale, prospective, observational study of zinc supplementation therapy for chemotherapy-induced dysgeusia. Most patients undergoing chemotherapy with dysgeusia were zinc-deficient and showed a dose-dependent increase in serum zinc levels with zinc supplementation. However, there was no statistically significant improvement in dysgeusia in all assessment scores in the zinc acetate hydrate group, which showed the highest increase in serum zinc levels compared with the no intervention group. On the other hand, there was a statistically significant improvement in dysgeusia in the polaprezinc group compared with the no intervention group when assessed using the STTA criteria. Zinc therapy is commonly used for chemotherapy-induced dysgeusia. Importantly, elevated plasma zinc levels reportedly improved dysgeusia in patients without malignancy-related dysgeusia [21, 29]. However, the present study showed no significant correlation between elevated serum zinc levels and improvement in chemotherapy-induced dysgeusia. There was also no clinically significant improvement in QOL. This may be because chemotherapy-induced dysgeusia is caused by several factors, such as the neurotoxic effects of chemotherapy, loss of sense of smell, secretion of chemotherapy drugs and metabolites into the saliva, and taste bud dysfunction caused by inflammatory cytokines produced by cancer, in addition to zinc deficiency and abnormal growth and repair of taste bud cells [7, 30–32].

Fujii et al. [17] and Mizukami et al. [22] reported that polaprezinc improved chemotherapy-induced dysgeusia in a single-center, retrospective study. The present study also showed a significant improvement in taste using STTA score in the polaprezinc group as well as a trend toward improvement in CTCAE, VAS, and CiTAS scores. Although it is not clear why dysgeusia improved in the polaprezinc group, our results suggest the involvement of factors other than zinc supplementation via polaprezinc administration. Polaprezinc contains 78% l-carnosine as well as a varied zinc content. Carnosine is an endogenous dipeptide composed of *β*-alanine and l-histidine. Carnosine is present in many organisms, such as birds, fish, and mammals, including humans. It is abundantly present in skeletal muscle, and it is also observed in the stomach, kidneys, cardiac muscle, and brain. Carnosine has various advantageous characteristics, such as antiglycation and antioxidant properties, hydroxyl radical scavenging, maintenance of pH balance, enhanced wound healing, and chelation of metals including divalent zinc ion (Zn^2+^) and bivalent copper ion (Cu^2+^) [33–39]. Yehia et al. [40] reported that l-carnosine improved oxaliplatin-induced peripheral neuropathy in oxaliplatin-treated cancer patients. In that study, the anti-inflammatory effects of l-carnosine were confirmed by its ability to reduce nuclear factor kappa-light-chain-enhancer of activated B cells and tumor necrosis factor-alpha, and it showed antioxidant effects by enhancing nuclear factor-2 erythroid related factor-2 and reducing levels of malondialdehyde, showing anti-apoptotic effects by reducing caspase-3. Furthermore, carnosine synthase activity was 50- to 100-fold higher in the olfactory epithelium than in brain structures [41, 42]. Zinc-l-carnosine may have contributed to anti-inflammatory effects, enhancing healing of taste bud cells, protection from chemotherapy-induced neurotoxicity, and improvement of olfactory loss due to the added effects of carnosine. It remains unclear whether carnosine contributes to the improvement of dysgeusia.

In the present study, polaprezinc showed greater efficacy in chemotherapy-induced dysgeusia than zinc acetate hydrate. This may be due to limitations, such as a possible selection bias as this was not a randomized trial. In Japan, many patients with chemotherapy-induced dysgeusia do not undergo intervention owing to a lack of established treatment approaches for chemotherapy-induced dysgeusia. Therefore, we included the no intervention group as the observation group in the present study. However, as the therapeutic approach for dysgeusia was based on the decision of the attending physician, a certain degree of bias in the choice of treatment method cannot be denied. A greater understanding of these background characteristics and a validation study with a placebo control is warranted to understand the taste-improving effects of polaprezinc. To the best of our knowledge, no large-scale prospective study investigated chemotherapy-induced dysgeusia and appropriate intervention methods. Therefore, this study was designed as an exploratory investigation without blinding. The findings of the present study provide valuable data for future placebo–control trials.

Four assessment tools were used as taste assessment methods in the present study. All of the scales were subjective assessments using self-reporting in which the patients answered a questionnaire. Established objective methods include electrogustometry [43], filter paper disc method [44], and whole-mouth gustatory test [45], which are used in otolaryngology. Although these objective indices are effective in evaluating the detection and cognitive thresholds of taste, they cannot be used to assess subjective symptoms, such as hallucinations and cacophony, which are commonly observed in cancer patients undergoing chemotherapy. Chemotherapy-induced taste changes can be described as a complex experience that encompasses many factors, including changes in smell, touch, and preference. Therefore, the patient’s subjective symptoms are important. The scale used in the present study is a widely used, reliable, and valid tool for assessing patients’ subjective symptoms.

Based on the current study’s findings, we plan to conduct a double-blinded, randomized, controlled study using placebo, polaprezinc, and carnosine to evaluate the efficacy of polaprezinc and carnosine in chemotherapy-induced dysgeusia and to elucidate factors apart from zinc that may be involved in chemotherapy-induced dysgeusia. Taste evaluation methods such as electrogustometry, filter paper disc method, and whole-mouth gustatory test will also be considered.

## Conclusions

In the present study, administration of polaprezinc or zinc acetate hydrate increased serum zinc levels; however, there was no significant correlation between the degree of serum zinc elevation and improvement of dysgeusia. Dysgeusia caused by chemotherapy may be a complex condition that involves factors other than zinc depletion.

Supplementary information.

## Ethics approval

All procedures were conducted in accordance with the ethical standards of the Helsinki Declaration of 1964 and its later versions. All patients received information about the study in written form and provided informed consent before enrollment. The study design and protocol were approved by the Institutional Review Board of Hokkaido University Hospital (approval number: 019–0248).

**Consent to participate**

Informed consent was obtained from all individual participants included in the study.

**Consent for publication**

Patients signed informed consent regarding publishing their data.

## Conflict of interest

The authors declare no competing interests.

## Supplementary Information

Below is the link to the electronic supplementary material.

Supplementary file1 (DOCX 39 KB)

## Data Availability

The datasets during and/or analyzed during the current study are available from the corresponding author on reasonable request.
