# Supplementary material for: Multicenter, prospective, observational study of chemotherapy-induced dysgeusia in gastrointestinal cancer
Source: Support Care Cancer. 2022 Mar 15;30(6):5351–9. doi: 10.1007/s00520-022-06936-4 (PMC9046346; doi:10.1007/s00520-022-06936-4)
Supplement: Supplementary file 1 — Supplementary file1 (DOCX 39 KB) [file 520_2022_6936_MOESM1_ESM.docx]

Table 1 Patient characteristics before zinc supplementation

| **n (%)** |  | **No intervention**  **(n = 53)** | **Zinc acetate hydrate**  **(n = 60)** | **Polaprezinc**  **(n = 59)** |
| --- | --- | --- | --- | --- |
| Sex | Male  Female | 25 (47)  28 (53) | 42 (70)  18 (30) | 36 (61)  23 (39) |
| Age | Median (range) | 70 (27–86) | 70 (42–81) | 67 (40–79) |
| Body mass index | Median (range) | 21.0 (14.6–33.5) | 21.9 (16.2–30.7) | 21.9 (15.4–30.5) |
| ECOG PS | 0  1  2 | 28 (53)  24 (45)  1 (2) | 37 (62)  23 (38)  0 (0) | 35 (59)  24 (41)  0 (0) |
| Professional oral care^*^ | Yes  No | 18 (34)  35 (66) | 26 (43)  34 (57) | 20 (34)  39 (66) |
| Mouth rinses | Yes  No | 27 (51)  26 (49) | 39 (65)  21 (35) | 32 (54)  27 (46) |
| Duration of dysgeusia (day) | Median (range) | 36 (1–1,933) | 23 (1–1,167) | 29 (1–3,697) |
| Cancer site | Esophagus  Gastric  Colorectal  Pancreatic  Biliary tract  Others | 2 (4)  15 (28)  26 (49)  6 (11)  4 (8)  0 (0) | 1 (2)  12 (20)  28 (47)  12 (20)  6 (10)  1 (2) | 0 (0)  14 (24)  30 (51)  7 (12)  7 (12)  1 (2) |
| Chemotherapy drugs | Platinum  Fluoropyrimidines  Taxanes | 28 (53)  38 (72)  10 (19) | 30 (50)  48 (80)  8 (13) | 35 (59)  45 (76)  7 (12) |
| Number of pretreatments | 0  1  ≥2 | 37 (70)  11 (21)  5 (9) | 41 (68)  17 (28)  2 (3) | 41 (69)  12 (20)  6 (10) |
| Serum zinc (μg/dL) | Median (range) | 68 / (29–113) | 64 / (43–109) | 66 (25–109) |
| Hb (g/dL) | Median (range) | 11.2 (8.3–16.1) | 11.5 (8.1–16.4) | 11.0 (8.0–16.2) |
| Alb (g/dL) | Median (range) | 3.6 (2.1–4.5) | 3.7 (2.6–4.5) | 3.5 (2.4–4.5) |
| Ferritin (ng/mL) | Median (range) | 129.5 (3.7–1855) | 155.1 (7–1343) | 97.2 (4–1051) |
| Vit B_12_ (pg/mL) | Median (range) | 457 (110–1500) | 478 (108–1500) | 513 (144–1500) |

*Oral care delivered by dental professionals

Alb, albumin; Hb, hemoglobin; ECOG PS, Eastern Cooperative Oncology Group performance score

Table 2 The results of QOL assessment using QOL-ACD

| Average (95%CI) | **No intervention**  **(n = 51)** | **Zinc acetate hydrate　(n = 52)** | | | **Polaprezinc　(n = 57)** | | | |  |
| --- | --- | --- | --- | --- | --- | --- | --- | --- | --- |
|  |  |  | ***P value***^†^ | |  | | ***P value***^†^ | |  |
| Daily activity | −1.41 (−2.80–−0.02) | −0.50 (−1.77–0.77) | | 0.686^*^ | | 0.00 (−1.37–1.37) | | 0.414^*^ | |
| Physical condition | 0.08 (−1.11–1.27) | 0.46 (−0.64–1.57) | | 0.938^*^ | | 1.12 (−0.02–2.26) | | 0.454^*^ | |
| Psychological condition | −0.63 (−1.66–0.41) | 0.19 (−0.45–0.84) | | 0.360^*^ | | 1.12 (0.10–2.14) | | 0.049^*^ | |
| Social attitude | 0.14 (−0.91–1.19) | 0.63 (−0.27–1.54) | | 0.817^*^ | | 0.54 (−0.42–1.51) | | 0.921^*^ | |
| Face scale | −0.31 (−0.60–−0.02) | 0.19 (−0.04–0.42) | | 0.021^*^ | | 0.04 (−0.21–0.28) | | 0.190^*^ | |
| Total | −2.14 (−5.50–1.22) | 0.98 (−1.64–3.60) | | 0.237^*^ | | 2.82 (−0.36–6.01) | | 0.083^*^ | |

^†^vs no intervention, ^*^ Steel's multiple comparison test

QOL, quality of life; QOL-ACD, quality-of-life questionnaire for cancer patients treated with anticancer drugs; CI, confidence interval

Online Resource 1 Participating institutes and number of enrolled patients

|  | Number of patients enrolled  (No intervention/zinc acetate hydrate/polaprezinc) |
| --- | --- |
| Institute A | 36 (11/15/10) |
| Institute B | 36 (20/7/9) |
| Institute C | 34 (8/14/12) |
| Institute D | 14 (5/5/4) |
| Institute E | 11 (1/1/9) |
| Institute F | 10 (3/6/1) |
| Institute G | 9 (6/1/2) |
| Institute H | 7 (1/5/1) |
| Institute I | 7 (2/1/4) |
| Institute J | 5 (1/2/2) |
| Institute K | 4 (2/2/0) |
| Institute L | 4 (0/0/4) |
| Institute M | 1 (0/1/0) |
| Institute N | 1 (0/0/1) |
| Institute O | 1 (0/0/1) |
| Institute P | 0 (0/0/0) |
| Institute Q | 0 (0/0/0) |
| Total | 180 (60/60/60) |

Online Resource 2 Changes in taste perception using VAS score

| Average (mm) (95% CI) | **No intervention**  **(n = 52)** | **Zinc acetate hydrate (n = 52)** | | **Polaprezinc (n = 57)** | |
| --- | --- | --- | --- | --- | --- |
|  |  |  | ***P* value**^†^ |  | ***P* value**^†^ |
| Baseline | 35.6  (29.1–42.1) | 44.5  (38.2–50.8) | 0.106^*^ | 37.6  (30.6–44.7) | 0.931^*^ |
| 12 weeks | 29.3  (22.7–35.8) | 38.4  (30.9–46.0) | 0.170^*^ | 26.9  (20.1–32.7) | 0.800^**^ |
| **Change from baseline at 12 weeks** | **6.6**  **(1.0**–**12.1)** | **6.1**  **(−1.6**–**13.7)** | **0.994^*^** | **10.8**  **(4.7**–**16.9)** | **0.669^*^** |

^†^vs no intervention, ^*^ Steel's multiple comparison test

VAS, Visual Analog Scale; CI, confidence interval

Online Resource 3 Changes in taste perception using CiTAS score

| Average (95% CI) | **No intervention**  **(n = 51)** | **Zinc acetate hydrate　(n = 52)** | | **Polaprezinc　(n = 57)** | |
| --- | --- | --- | --- | --- | --- |
|  |  |  | ***P value***^†^ |  | ***P value***^†^ |
| Decline in basic taste | 0.16 (−0.04–0.37) | 0.36 (0.05–0.67) | 0.359^*^ | 0.47 (0.26–0.68) | 0.102^*^ |
| Discomfort | −0.03 (−0.21–0.15) | 0.12 (−0.09–0.33) | 0.689^*^ | 0.22 (0.04–0.40) | 0.109^*^ |
| Phantogeusia and parageusia | 0.03 (−0.16–0.22) | 0.13 (−0.21–0.47) | 0.968^*^ | 0.21 (−0.06–0.48) | 0.445^*^ |
| General taste alterations | 0.08 (−0.09–0.26) | 0.35 (0.04–0.65) | 0.205^*^ | 0.44 (0.18–0.71) | 0.089^*^ |

^†^vs no intervention, ^*^ Steel's multiple comparison test

CiTAS, Chemotherapy-induced Taste Alteration Scale; CI, confidence interval

Online Resource 4 The results of logistic regression analysis for STTA score

|  |  |  | **Multivariate analysis** | |
| --- | --- | --- | --- | --- |
|  |  |  | Odds (95%CI) | *P value* |
| Age | Median (range) | 68.0 (27–86) | 0.969 (0.930–1.010) | 0.139 |
| Sex | Male  Female | 105 (60)  70 (40) | 1.775(0.813–3.876) | 0.150 |
| ECOG PS | 0  1  2 | 101 (58)  73 (42)  1 (1) | 1.481 (0.686–3.200)  - | 0.312  - |
| Body mass index | Median (range) | 21.7 (14.6–33.5) | 1.060 (0.946–1.188) | 0.315 |
| Dysgeusia treatment | No intervention  Zinc acetate hydrate  Polaprezinc | 56 (32)  60 (34)  59 (34) | 0.975 (0.303–2.712)  3.196 (1.283–7.962) | 0.966  **0.013** |
| Professional oral care*** | Yes  No | 66 (38)  109 (62) | 1.700 (0.750–3.850) | 0.204 |
| Mouth rinses | Yes  No | 101 (58)  74 (42) | 1.337 (0.608–2.939) | 0.470 |
| Duration of dysgeusia (days) | Median (range) | 29 (1–3697) | 1.001 (0.999–1.001) | 0.856 |
| Cancer site | Esophagus  Gastric  Colorectal  Pancreatic  Biliary tract | 3 (2)  42 (24)  86 (49)  25 (14)  17 (10) | -  1.271 (0.058–28.05)  1.112 (0.056–21.89)  1.361 (0.056–33.15)  1.013 (0.041–24.89) | -  0.879  0.944  0.850  0.994 |
| Chemotherapy drugs | Platinum  Fluoropyrimidines  Taxanes | 95 (54)  133 (76)  25 (14) | 0.704 (0.301–1.649)  2.068 (0.706–6.058)  2.510 (0.592–10.65) | 0.439  0.170  0.222 |
| Change in serum zinc level (μg/dL) | Median (range) | 78 (37–249) | 1.001 (0.989–1.013) | 0.968 |

STTA, Scale of Subjective Total Taste Acuity; ECOG PS, Eastern Cooperative Oncology Group performance score; CI, confidence interval
